# Impact of Left Ventricular Assist Devices on the Quality of Life and Functional Outcomes in Advanced Heart Failure: A Comprehensive Review

**DOI:** 10.7759/cureus.88527

**Published:** 2025-07-22

**Authors:** Syed Maaz Shah, Hira Saleem, Vaisnavy Govindasamy, Amandeep Rathi, Neha Haris, Ramsha Ali, Lakshmi Devi, Layal E Omaruddin, Krishna S Pavuluri, Anit M Joseph, Abdullah Tariq

**Affiliations:** 1 Department of Internal Medicine, Kansas City University College of Osteopathic Medicine, Kansas City, USA; 2 Department of Internal Medicine, Houston Methodist Research Institute, Houston, USA; 3 Department of Medicine, James Cook University Hospital, South Tees NHS Trust, Middlesbrough, GBR; 4 Department of Internal Medicine, Government Medical College, Amritsar, Amritsar, IND; 5 Department of Internal Medicine, Medcare Hospital, Sharjah, ARE; 6 Department of Medicine and Surgery, Peoples University of Medical and Health Sciences for Women, Nawabshah, PAK; 7 Department of Internal Medicine, Manchester University NHS Foundation Trust, Manchester, GBR; 8 Department of Internal Medicine, Royal College of Surgeons in Ireland - Bahrain, Busaiteen, BHR; 9 Department of Internal Medicine, Sri Ramachandra Medical College and Research Institute, Chennai, IND; 10 Department of Internal Medicine, Victoria Hospital - Kirkcaldy, NHS Fife, Kirkcaldy, GBR; 11 Department of General Practice, Divisional Headquarters Teaching Hospital - Mirpur, Mirpur, PAK

**Keywords:** 6 minute walk test, bridge to transplant, complications, destination therapy, functional recovery, heart failure, left ventricular assist device, lvad, psychological support, quality of life

## Abstract

Managing advanced heart failure (HF) remains a major clinical challenge, especially for patients who no longer respond to conventional treatment options, or are not candidates for heart transplantation. In such cases, left ventricular assist devices (LVADs) have become an important option, offering mechanical support that has the potential to extend life and improve daily functioning. With ongoing advancements in technology, there is growing interest in understanding how LVADs impact not just survival, but also patients' quality of life (QoL) and overall recovery.

This narrative review explores the role of LVAD therapy in improving functional status and QoL in patients with advanced HF. It draws on clinical research from the past 15 years to examine physical, psychological, and social outcomes, as well as the challenges and complications associated with this treatment. A structured PubMed search was performed using predefined MeSH terms and keywords related to LVADs. After removing duplicates, 68 articles were identified. Eleven reviewers screened titles and abstracts, followed by full-text review and data extraction. Studies were included if they involved adults, were conducted in the past 15 years, and used clinical or observational study designs. Non-English, pediatric, and pregnancy-related studies were excluded. A total of 44 studies met the criteria. LVADs were associated with improvements in physical function, exercise capacity, and QoL. However, patients often faced psychological stress and medical complications. Better pre-implant health and strong support systems contributed to more favorable outcomes. LVADs offer meaningful improvements in both survival and QoL, but holistic, patient-centered care remains key to optimizing outcomes.

## Introduction and background

As of 2021, approximately 55 million people were diagnosed with heart failure (HF) worldwide - an overwhelming number that reflects a steady increase from 25.4 million in the 1990s. HF is a complex clinical syndrome characterized by a decreased ability of the heart to pump and/or fill with blood. As the condition becomes more common, innovative treatments like the left ventricular assist device (LVAD) are gaining popularity. The primary objective of this narrative review is to compile data from studies conducted over the last 15 years that focus on the functional outcomes and quality of life (QoL) of LVAD patients [1,2]. The outstanding research question is: How does LVAD therapy affect the general QoL and functional ability of HF patients?

HF treatment has traditionally focused on a combination of medications and lifestyle changes to help manage symptoms and slow the progression of the disease. For those with end-stage HF, advanced therapies such as LVAD implantation, a form of mechanical circulatory support (MCS), are specifically recommended for patients with heart failure with reduced ejection fraction (HFrEF) (≤40%) who have failed to respond to traditional treatments, including renin-angiotensin-aldosterone system (RAAS) antagonists (i.e., ramipril), beta blockers (i.e., metoprolol), and cardiac resynchronization therapy (CRT) [3]. LVADs were originally developed as a bridge to transplantation (BTT); however, they are now employed as a suitable modality for patients who are not eligible for heart transplantation due to advanced age and significant comorbidities (i.e., myocardial dysfunction, valve abnormalities, or rhythm disturbances). As an alternative to a heart transplant, patients with end-stage HF have strongly benefited from an LVAD implant, with a one-year survival of 84%, and substantial improvement in QoL was noted as early as one month post-surgery [4,5]. This category of LVAD indication is termed destination therapy (DT). The Randomized Evaluation of Mechanical Assistance for the Treatment of Congestive Heart Failure (REMATCH) trial played a key role in establishing DT by demonstrating that LVADs, such as the HeartMate VE, could improve survival and QoL in non-transplant recipients [6]. 

**Figure 1:**
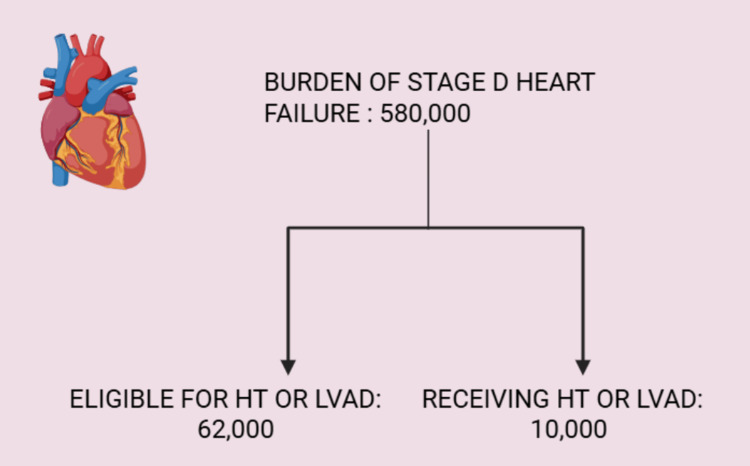
Burden of stage-D heart failure The given statistics are according to the USA population, as reported by the Journal of the American Heart Association [7]. Image credits: Neha Haris; created in BioRender.com HT: Heart transplant; LVAD: Left ventricular assist device

According to the Journal of the American Heart Association, in the United States alone, around 580,000 people are believed to have Stage-D HF, of whom 62,000 are eligible for LVAD or heart transplantation, but only 10,000 receive such care. It is understandable that there is a limitation to heart transplantation due to the limited number of donor hearts, but such a limitation does not exist with LVAD (Figure 1) [7]. The authors of this review will also explore the impact of LVAD on the incidence of mortality in the adult population (>18 years), along with adverse events following implantation. As the use of LVADs grows, there is a clear need for a comprehensive review of their benefits, risks, and long-term effects. By examining current literature, this review can help refine clinical decision-making, improve patient education, optimize long-term care strategies, and guide future research. 

LVADs enhance QoL while reducing symptoms of HF, as well as improving patient mobility and increasing physical capability. Various patients have recorded enhanced performance in their activities of daily living (ADLs), together with increased self-reliance [8,9]. Milestones achieved at the three-month mark post-surgery were optimal peak oxygen uptake and re-classification of HF to New York Heart Association (NYHA) Class I or II. LVADs have shown improvement in QoL and functional capacity; however, LVAD-related complications have shown a negative effect on long-term prognosis [10,11]. Treatment with LVADs can cause complications including bleeding (26.9% rural vs. 24.7% urban, p = 0.37), driveline infections (26.9% rural vs. 24.7% urban, p = 0.37), neurological events (13.5% rural vs. 7.9% urban, p = 0.83), and right ventricular dysfunction (3.9% vs. 6.7%, p = 0.74) [12-14], that could negate patient perception of benefits, although these complications might not occur for all patients. Apart from the consequences listed above, LVADs are associated with a higher risk of rehospitalization and device-related infections (both p < 0.05) in patients with psychiatric conditions such as alcohol abuse, drug use, depression, and other major psychiatric illnesses - with the important exception of narcotic dependence, as per a review registered in the Interagency Registry for Mechanically Assisted Circulatory Support (INTERMACS) between 2008 and 2017 [15]. In a contradictory review, it was demonstrated that LVADs lead to lower rates of depressive symptoms, i.e., reduced sleep and social withdrawal, along with anxiety related to device management [5,16]. 

The focus of this study is to assess LVAD use while also discussing parameters such as the benefits of LVAD therapy, common adverse events, mental health consequences, and the financial burden on healthcare of this implant as compared to standard therapy. Elaborating further on the financial perspective, if we were to make a comparison with their medical alternatives, the implantation of an LVAD remains above the baseline parameter, with an incremental cost-effectiveness ratio (ICER) of £54,748 per QALY (Quality-Adjusted Life Year) [14].

The aim of this review is to outline factors that have an overall impact on a patient's health following LVAD surgery. We screened papers relevant to our primary aim of compiling surgical and psychological complications listed in the literature, along with a comprehensive outlook on the accumulated effect of these outcomes on morbidity and mortality through tools of functional recovery such as the six-minute walk test (6MWT), VO_2_ peak, etc. Additionally, this review examines the cost-effectiveness and healthcare utilization of LVAD implantation in both men and women. The subsequent portions of this paper will discuss the methods used in current studies, present key results, and draw conclusions to enhance our understanding of LVAD therapy by highlighting principal discoveries and identifying areas that need further study to improve patient-centered care.

## Review

Methods

We utilized search engines such as PubMed, EMBASE, Cochrane, and Rayyan. The search terms included combinations of keywords such as "heart transplantation," "left ventricular assist devices," "LVAD," "heart failure," "exercise capacity," "quality of life," "6-minute walk test (6MWT)," and "psychological effects." We focused on peer-reviewed original research articles, meta-analyses, and systematic reviews while excluding case series and case reports. The 6MWT is a widely recognized tool for assessing functional capacity in patients with advanced HF, with cardiopulmonary exercise testing (CPET) being another validated tool. A few of the outcome measures included in this study are questionnaires like the EQ-5D-5L and Kansas City Cardiomyopathy Questionnaire (KCCQ), Functional Independence Measure (FIM) scores, etc. We focused on peer-reviewed original research articles, systematic reviews, and retrospective and prospective cohort studies. Case reports and small case series were generally excluded. The quality of the studies included has been assessed based on their sample size, methodology, and relevance to this review's objectives. However, given the narrative nature of this review, a formal quality assessment tool could not be applied. 

Key findings

LVADs have shown significant improvements in QoL, functional capacity, and rate of survival for patients with advanced HF who are ineligible or awaiting transplantation [5,6]. Across the cited studies, LVADs have been associated with enhanced cardiac output, reduced HF symptoms, and improved functional capacity, leading to better exercise tolerance and reduced hospital admissions. Patients have shown improved 6MWT performance sustained up to 24 months, with enhanced peak VO₂ levels, though below age-adjusted norms [17]. Inpatient cardiac rehabilitation (CR) after LVAD implantation is linked to improved functional independence and reduced 30-day readmission rates, as measured by significant improvements in the FIM, a validated scale that assesses a patient’s ability to perform ADLs and their level of disability [18,19]. QoL benefits include better physical function and fewer hospitalizations, though patients often face psychological challenges requiring ongoing support. However, while these physical benefits are evident, patients still experience substantial psychological challenges, including emotional stress from self-care demands, lifestyle changes, pain, and medication management [20,21]. Favorable outcomes are associated with better pre-implant health and strong social support. Technological advancements have made LVADs a viable alternative to heart transplantation, offering comparable long-term survival and meaningful improvements in functional status [22].

**Figure 2:**
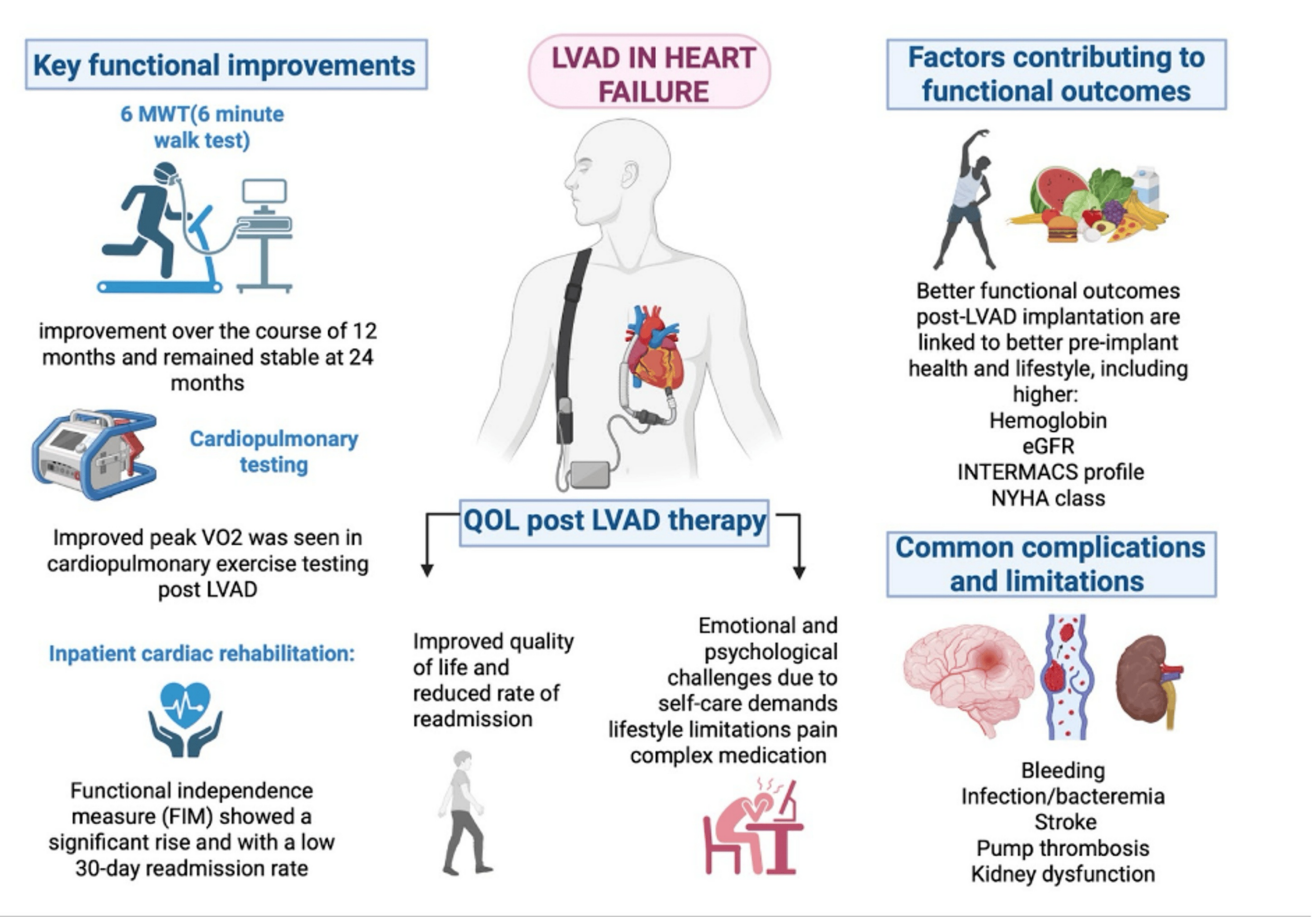
Highlights of left ventricular assisted devices in heart failure Image credits: Amandeep Rathi; created in BioRender.com LVAD: Left ventricular assisted devices; QoL: Quality of life; eGFR: Estimated glomerular filtration rate; INTERMACS: Interagency registry for mechanically assisted circulatory support; NYHA: New York Heart Association

Table 1 provides a comprehensive summary of key findings from the studies included in this review, organized into major clinical themes based on their relevance to LVAD therapy, along with their associated supporting evidence [17-22]. These findings are visually summarized in Figure 2, which highlights the key findings of LVAD therapy in HF, such as functional gains and enhanced QoL - factors contributing to functional outcomes - alongside the main challenges, including device-related complications and psychological burden. 

**Table 1:** Research question with key findings and supporting evidence LVADs: Left ventricular assisted devices; QoL: Quality of life; 6MWT: Six-minute walk test; FIM: Functional independence measure; eGFR: Estimated glomerular filtration rate; INTERMACS: Interagency registry for mechanically assisted circulatory support; NYHA: New York Heart Association

Research Question	Key Finding(s) Results	Supporting Evidence (Examples)
Key functional improvements	6MWT: Functional gains measured by 6MWT studies have shown improvement over the course of 12 months and remained stable at 24 months. Cardiopulmonary testing: Improved peak VO₂ was seen in cardiopulmonary exercise testing post-LVAD, but the functional gains were lower than the age-adjusted norms. Inpatient cardiac rehabilitation: Studies have shown that, following inpatient rehabilitation, the FIM significantly increased and was associated with a low 30-day readmission rate.	6MWT & cardiopulmonary testing [17]; Inpatient cardiac rehabilitation [18,19]
QoL post-LVAD therapy (i.e., physical and psychological aspects)	LVADs improved exercise capacity (subjective assessment). Left ventricular assist devices have shown promise in improving QoL and decreasing hospital readmissions. Mental health: LVAD patients face emotional and psychological challenges due to self-care demands, lifestyle limitations, pain, and complex medication management. Significant mental health effects require psychological support and coping strategies.	Exercise capacity & QoL [20], hospital readmission [21], mental health [15,22]
Common complications and limitations experienced by LVAD recipients	LVADs, while beneficial, are associated with several complications. These include bleeding, infection/bacteremia, stroke, and pump thrombosis. Kidney dysfunction is common.	Soliman et al. (2018) [23]; Castagna et al. (2017) [24]
Patient and clinical factors contributing to better functional outcomes post-implantations	Better functional outcomes post-LVAD implantation are linked to better pre-implant health, including higher hemoglobin, eGFR, INTERMACS profile, and NYHA class. Respiratory failure before or after implantation is associated with worse 1-year mortality. Additionally, strong social support correlates with improved QoL, even after adjusting for fatigue.	Miller et al. (2019) [11]; Kirklin et al. (2013) [25]
Technological advancements such as LVADs leading to better outcomes	As the number of suitable heart donors, experienced medical centers, and patient comorbidities place restrictions on the feasibility of cardiac transplantation, conceptual models have emerged that integrate the main determinants of LVAD utilization, such as insurance coverage, technology, market provider for the device and patient factors. Moreover, technological advancements such as LVADs have proven to be a viable alternative - as destination therapy or as a bridge to transplantation - since they have demonstrated similar long-term survival rates and significant improvements in health-related QoL and functional status.	Miller et al. (2019) [11]; Ward et al. (2017) [26]

**Figure 3:**
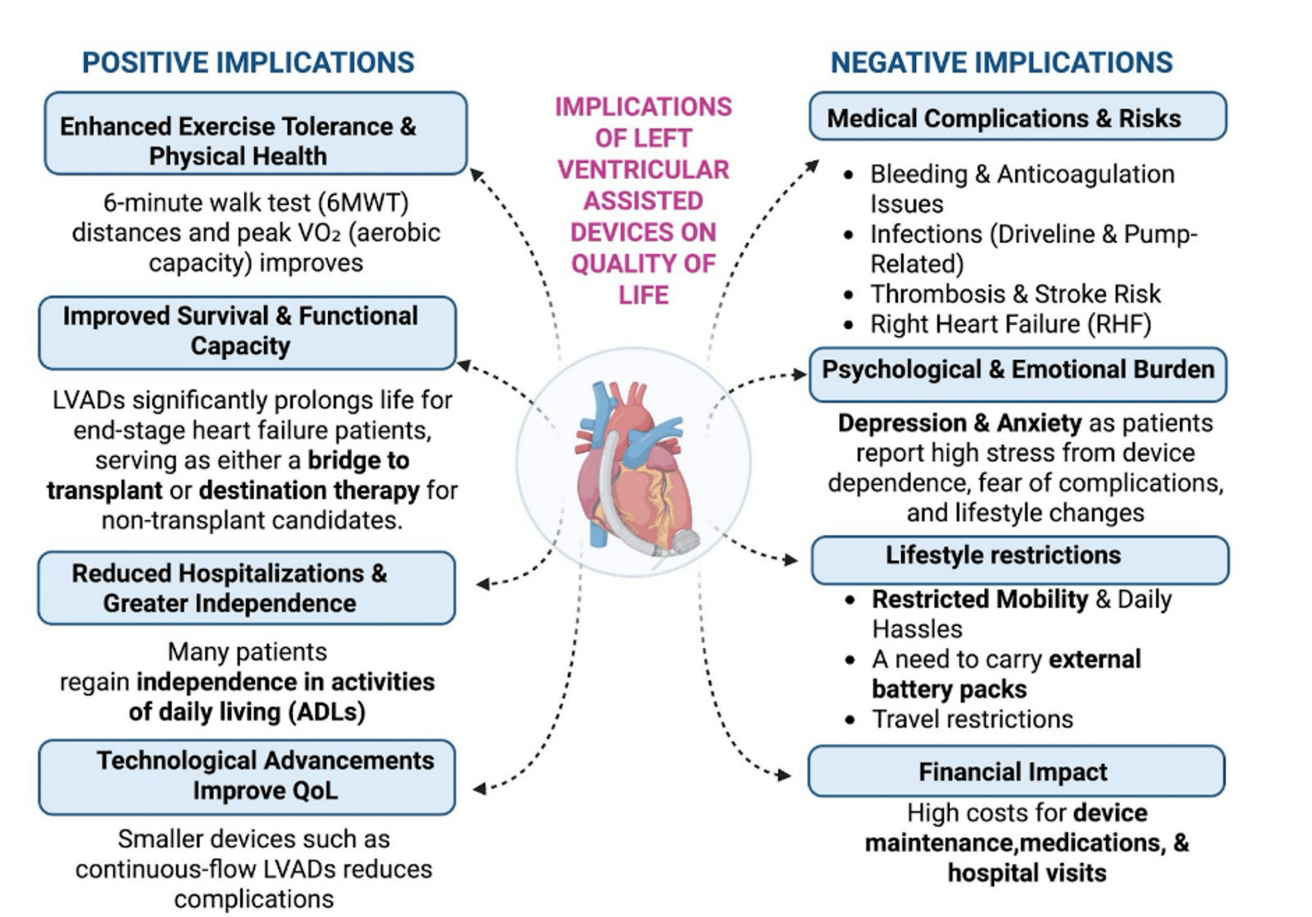
Implications of left ventricular assisted devices on quality of life Image credits: Amandeep Rathi; created in BioRender.com LVAD: Left ventricular assisted devices; ADLs: Activities of daily living

Additionally, long-term LVAD management is complicated by persistent medical risks, such as device-related complications, thrombosis, infection, bleeding, and the need for lifelong anticoagulation, as well as psychological and emotional implications. Moreover, high costs and mobility restrictions significantly impact patients' day-to-day life [27-29]. Figure 3 summarizes the positive and negative implications associated with LVAD therapy. These implications underscore the need for comprehensive care strategies that address both the physical and psychosocial dimensions of living with an LVAD. 

Discussion

The results from this study support earlier findings, confirming that LVAD therapy improves physical health and reduces hospital readmissions [20,21]. These results are consistent with previous research on the benefits of LVADs in treating end-stage HF. In terms of complications, the issues we identified - including bleeding and kidney dysfunction - mirror those highlighted by Castagna et al. [24] and Muslem et al. [27], emphasizing that, while LVAD therapy can improve survival, the associated risks still need careful management.

As previously mentioned, living with an LVAD can lead to significant psychological challenges. Addressing the mental health impact of LVADs requires more than just medical intervention; it calls for a holistic and personalised approach. From the outset, patients should be screened for existing psychological or cognitive issues, with regular assessments throughout their journey to identify any emerging concerns. Emotional support is essential, whether through relaxation techniques, counseling, or simply having someone to talk to. Education also plays a crucial role by helping patients understand their device, adjust to lifestyle changes, and regain the confidence to return to their daily routine or work. Above all, continuous psychological support tailored to each individual's needs ensures that patients feel supported and never alone in their recovery journey [15-17].

Elaborating further on the physical aspects of LVAD therapy is the improvement in the 6MWT following LVAD implantation. The 6MWT is a widely recognized tool for assessing functional capacity in patients with advanced HF, including those supported by LVADs. Numerous studies have highlighted significant improvements in walking distance following LVAD implantation [24]. A notable study published in the Journal of Heart and Lung Transplantation analyzed data from the INTERMACS registry, which included 9,690 patients with continuous-flow LVADs implanted across 143 institutions between April 2008 and March 2014. Of these, 1,738 patients were ambulatory at baseline (AMB group), while 4,888 patients were non-ambulatory (Non-AMB group) and assigned a baseline 6MWD of zero feet. The study found that non-AMB patients were generally younger, more acutely ill, and more likely to be listed for transplantation. Despite their initial limitations, the 6MWD improved significantly in both groups by the third month post-implantation and remained stable up to 24 months. These findings underscore the potential for substantial functional recovery, even among the most critically ill patients [30].

Moreover, a systematic review and meta-analysis found a weighted mean difference (WMD) improvement of 60.06 meters in 6MWT distance for LVAD patients who participated in exercise-based cardiac rehabilitation (EBCR), compared to those receiving standard therapy (95% CI: 22.61-97.50, p = 0.002) [31]. Long-term follow-up studies showed that the functional improvements observed following rehabilitation were sustained over time, with some patients maintaining these gains for at least 12 months after implantation.

However, despite these promising outcomes, not all LVAD recipients achieve optimal recovery. Research published in the American Journal of Cardiology has shown that certain patients, even with LVAD support, continue to face exercise intolerance. Diabetes mellitus and elevated right atrial pressure were identified as significant predictors of poor performance in the 6MWD following LVAD implantation, underscoring the influence of comorbidities and hemodynamic factors on functional recovery [32].

The degree of improvement in 6MWD appears to be closely associated with adherence to rehabilitation programs, highlighting the importance of structured, continuous interventions to maximize patient outcomes. Tailored rehabilitation strategies, including individualized exercise prescriptions and early mobilization, could further enhance functional recovery in LVAD recipients.

CPET is another objective tool used to assess aerobic capacity, particularly peak oxygen uptake (VO₂ peak), in LVAD patients. Multiple studies have demonstrated significant improvements in VO₂ peak following CR. A meta-analysis encompassing 12 trials with 477 participants revealed a WMD of 1.64 mL/kg/min in VO₂ peak in those who underwent EBCR, compared to those receiving standard therapy [33].

Additionally, increasing LVAD pump speed has been shown to correlate with better exercise performance, including improved VO₂ peak, delayed onset of anaerobic threshold, and greater ventilatory efficiency [34]. A case study published in CJC Open described a patient who achieved significant gains in VO₂ peak after completing over 1,000 hours of exercise training [35].

The Rehab-VAD randomized controlled trial further supports these findings, reporting a 10% increase in VO₂ peak among LVAD patients who participated in a structured CR program post-implantation [18]. Despite these improvements, many LVAD patients still exhibit lower exercise tolerance compared to age-matched healthy individuals [36].

Moreover, LVAD patients often experience chronotropic incompetence - the inability of the heart to adequately increase its rate during physical activity - which can contribute to reduced exercise performance. This suggests that factors beyond cardiac output, such as autonomic dysfunction, may limit exercise capacity in this population [37].

Improvements in ventilatory efficiency, another key component measured in CPET, have also been shown to enhance overall functional performance in LVAD patients. Elevated dead space ventilation (V_D/V_T) - the fraction of each breath that does not contribute to gas exchange - during exercise has been identified as a potential marker for right ventricular to pulmonary artery uncoupling, which may explain the persistent exercise limitations despite LVAD support [38].

Numerous studies, such as one by Inyom et al. [39], have highlighted that based on questionnaires like the EQ-5D-5L (a standardized tool to measure health-related QoL) and the KCCQ (a 23-item self-administered tool used to assess the health status of patients with HF), patients post-LVAD implantation have shown significant improvement in exercise capacity and QoL. To further support this, a study by Goodwin et al. [20] showed that patients supported with LVAD experienced a significant increase in exercise capacity 6 to 12 months post-implantation.

Despite improvements in functional capacity, its implications on mental health have severely impacted health-related QoL. LVAD patients face emotional and psychological challenges due to self-care demands, lifestyle limitations, pain, and complex medication management. Individuals with high perceived stress experience worse depressive symptoms, fatigue, and coping difficulties. These significant mental health effects require psychological support and coping strategies. The influence of high social support in improving the relationship between stress and health-related QoL emphasizes the importance of a comprehensive plan to address psychosocial factors.

Besides its effects on mental health, a study by Shah and Qayed [40] revealed a fivefold increase in the risk of readmission due to gastrointestinal bleeding within 60 days following LVAD implantation. Ficinski et al. showed that, of those implanted with LVADs, patients with LVAD-related infections had higher costs and resource utilization, such as antibiotics and readmission [12]. An economic modeling study conducted by Saygın Avşar et al. showed that LVADs as DT were not found to be cost-effective based on the cost per QALY and the UK willingness-to-pay thresholds [14]. These findings emphasize the need for further evaluation and development of cost-effective pathways to LVAD therapy, based on device costs and post-LVAD complications. 

In summary, across the cited studies, there is a broad consensus that LVAD therapy leads to improvement in functional independence, health-related QoL, peak VO₂, and exercise capacity, although the magnitude of benefit varies according to comorbidities, psychosocial factors, and rehabilitation protocols [17-19,36-42]. 

The cited articles have certain limitations, such as their observational design, which limits inference of causality. There is significant heterogeneity in rehabilitation programs, follow-up duration, and sample size, which might make it difficult to compare and generalize findings. It has also been noted that gender-stratified results haven’t been reported, highlighting a potential research gap. Although individual case reports, such as the one by Loureiro Diaz et al. describing a patient on HeartMate 2 LVAD who received over 1,000 hours of CR over 10 years, show benefits of CR, and case series conducted by Hanke et al., which demonstrated the benefits of exchange to HeartMate 3 due to reduced pump thrombosis, show promising results, they cannot be generalized due to the small cohort size [35,43,44]. Nonetheless, such reports reveal potential avenues for future studies and decision-making in challenging situations.

Significance

The significance of this study lies in its dual focus on both the physical and psychological impacts of LVAD therapy. LVADs offer notable improvements in exercise capacity and QoL, with gains demonstrated by 6MWT and peak VO₂ levels maintained from 12 to 24 months post-implant [17]. LVADs provide MCS by assisting the heart in pumping blood, thereby enhancing cardiac output during physical activities. This mechanical assistance is crucial during both submaximal and maximal exercise intensities, as it helps meet the increased oxygen and nutrient demands of the body. Consequently, patients with LVADs often experience improved exercise tolerance and functional capacity, leading to better overall health outcomes. Additionally, these benefits contribute to a reduction in hospital readmissions, which is particularly significant for healthcare systems and cost management [21]. However, serious complications, including bleeding, infection/bacteremia, stroke, and pump thrombosis, remain common, and mental health challenges, such as emotional strain from self-care demands, lifestyle limitations, and medication complexity, may diminish the overall impact of physical improvements [20]. Developing evidence-based strategies, such as cognitive behavior therapy, integrated psychosocial care, or peer-supported models, may help to address the psychological challenges.

Studies have demonstrated that inpatient rehabilitation significantly enhances functional independence and ADLs in LVAD patients. Multiple studies have reported substantial gains in FIM scores following rehabilitation [4,41,42]. Similarly, a larger study involving 94 patients reported an average FIM increase of 28.4 points, with 74% of patients discharged directly home, underscoring the role of rehabilitation in facilitating independent living [19]. These findings collectively highlight the critical impact of structured rehabilitation programs on post-LVAD recovery.

Therefore, the introduction of LVADs has revolutionized the treatment of end-stage HF, significantly prolonging survival and offering a lifesaving option for patients awaiting transplantation or ineligible for one [43]. LVADs restore cardiac output, reduce congestion, and improve organ perfusion, resulting in better functional capacity, fewer hospitalizations, and increased independence. Their effectiveness is also influenced by pre-implant clinical and patient factors, including comorbidities and the degree of HF. Technological advancements - such as device miniaturization, enhanced biocompatibility, and innovations like Transcutaneous Energy Transfer Systems, which aim to provide energy transcutaneously, thereby eliminating the need for a percutaneous driveline and reducing driveline-associated infections - have made LVADs less invasive and reduced infection risk, thereby enhancing QoL [44]. These findings underscore the importance of integrating both physical and psychological care and continuing innovation to optimize outcomes for LVAD recipients.

Challenges and limitations

Although this narrative review provides valuable insights regarding the functional outcomes and adverse effects following LVAD therapy, it is limited by several factors. One limitation is that the data comes from published literature, which may introduce publication bias. The lack of a systematic methodology and the absence of pooled statistical or qualitative analysis may affect the interpretation and generalizability of the results. Additionally, the study primarily focuses on short-term outcomes, with most data extending only up to 24 months post-LVAD implantation. Longer-term follow-up, such as 5-year or 10-year follow-up, is necessary to determine whether improvements in physical function and QoL are sustained. Much of the available data also originates from specialized centers that have dedicated staff for LVAD therapy and multidisciplinary teams, which may not reflect the experiences of patients in broader, resource-limited settings or those with multiple comorbidities. In real-world settings, differences in patient demographics - such as lower socioeconomic status, limited access to follow-up care, and higher comorbidities - can lead to more complications and worse outcomes than those reported by specialized centers. Future research with patients from varying clinical settings and diverse demographics can help generalize the findings. 

Despite documented functional improvements post-implant, such as gains in peak VO₂, these values remain below age-adjusted norms, indicating incomplete physiological recovery [17]. Although challenges remain, such as the influence of comorbidities and individual variability in recovery, ongoing research supports the effectiveness of tailored rehabilitation strategies in maximizing recovery and QoL for LVAD recipients.

Furthermore, the literature reviewed does not encompass all dimensions of QoL that define functional outcomes. Significant mental health challenges - such as psychological stress, pain, and lifestyle limitations - persist, highlighting the importance of holistic care models that address both mental and physical outcomes [5]. This review also draws from observational studies, limiting the ability to establish causality, and, in some cases, small sample sizes reduce statistical power. Additionally, complications such as bleeding, infection, thromboembolic events, and kidney dysfunction continue to pose clinical risks [22]. Future studies should aim to include more diverse patient populations, employ longer follow-up periods, and explore technological innovations aimed at reducing complications while improving long-term outcomes.

Context

This study’s findings must be placed in the broader context of HF treatment, particularly as the demand for heart transplants continues to exceed the supply of available donors. LVADs are an important alternative for patients who are not candidates for a transplant, offering an opportunity to improve survival and QoL in this growing population [26]. In the broader scope of HF management, with the rising prevalence of advanced HF along with increased patient comorbidities, patients have shown an increased reliance on LVADs as a viable alternative to transplantation. This trend aligns with the current healthcare approach to provide personalized, technology-driven cardiac care.

In the broader scope of HF management, patients were found to have an increased reliance on LVADs as a viable alternative to transplantation. Due to the scarcity of suitable donors and the increasing prevalence of patient comorbidities, such outcomes align with the current healthcare approach to provide personalized, technology-driven cardiac care.

However, the risks associated with LVAD therapy - particularly the psychological and long-term physical complications - mean that healthcare providers need to take a comprehensive approach to care. Addressing these challenges and ensuring proper follow-up can help maximize the benefits of LVAD therapy while minimizing negative effects on patients’ mental and physical well-being. From a policy perspective, further research into the integration of psychosocial and rehabilitation services is essential to ensure more comprehensive outcomes. This study suggests that further research is needed focusing on mechanisms targeting LVAD speed, which could significantly improve left ventricular support during intense activities, including exercise capacity - an important functional outcome in patients on LVAD support. 

From a health system perspective, LVADs add a significant burden in costs for the device, the need for specialized multidisciplinary care teams, long-term follow-up care, and rehabilitation services. They also contribute to significant emotional distress for patients. To address these challenges, we need policy initiatives to expand funding for CR and mental health support services. Furthermore, enhancing insurance reimbursements and investing in infrastructure can help provide equitable access to a broader group of patients.

As part of the continued improvements, LVADs complement existing HF management, which includes medical therapy, cardiac devices, and transplantations, by serving as both a BTT and a potential DT.

This study suggests that further research is needed, focusing on mechanisms that would target LVAD dynamic pump speeds, where LVADs modulate flow rates based on real-time physiological demand, such as during exercise. This could significantly improve left ventricular support during intense activities, including exercise capacity, which is an important functional outcome in patients on LVAD support. Future research should also evaluate patients over longer terms, such as 5 or 10 years; focus on a structured plan for implementing mental health interventions (such as cognitive behavioral therapy and peer support group programs); and aim at enhancing the technology user interface to ensure greater patient usability and adherence.

## Conclusions

Through this study, we have found that LVADs bring significant improvement in both survival and QoL for patients with advanced HF. However, to enhance the results obtained, we must create unified, patient-centered metrics that evaluate physical parameters (such as peak VO₂ and 6MWT), psychological well-being (such as anxiety and depression scales), and health-related QoL questionnaires (such as EQ-5D-5L and KCCQ), adjusted for patient age and comorbidities. 

Additionally, we must address the need for improved LVAD technology, individualized treatment plans that incorporate patient-centered rehabilitation, and strategies to address the psychosocial challenges faced by LVAD recipients. Through these avenues of change, we can bring forth an enhanced living experience for patients.
