# Correction: Impact of Left Ventricular Assist Devices on the Quality of Life and Functional Outcomes in Advanced Heart Failure: A Comprehensive Review

**DOI:** 10.7759/cureus.c232

**Published:** 2025-07-25

**Authors:** Syed Maaz Shah, Hira Saleem, Vaisnavy Govindasamy, Amandeep Rathi, Neha Haris, Ramsha Ali, Lakshmi Devi, Layal E Omaruddin, Krishna S Pavuluri, Anit M Joseph, Abdullah Tariq

**Affiliations:** 1 Department of Internal Medicine, Kansas City University College of Osteopathic Medicine, Kansas City, USA; 2 Department of Internal Medicine, Houston Methodist Research Institute, Houston, USA; 3 Department of Medicine, James Cook University Hospital, South Tees NHS Trust, Middlesbrough, GBR; 4 Department of Internal Medicine, Government Medical College, Amritsar, Amritsar, IND; 5 Department of Internal Medicine, Medcare Hospital, Sharjah, ARE; 6 Department of Medicine and Surgery, Peoples University of Medical and Health Sciences for Women, Nawabshah, PAK; 7 Department of Internal Medicine, Manchester University NHS Foundation Trust, Manchester, GBR; 8 Department of Internal Medicine, Royal College of Surgeons in Ireland - Bahrain, Busaiteen, BHR; 9 Department of Internal Medicine, Sri Ramachandra Medical College and Research Institute, Chennai, IND; 10 Department of Internal Medicine, Victoria Hospital - Kirkcaldy, NHS Fife, Kirkcaldy, GBR; 11 Department of General Practice, Divisional Headquarters Teaching Hospital - Mirpur, Mirpur, PAK

This article has been corrected as a sentence in paragraph 8 of the Discussion section misattributed case study findings to the Journal of ExtraCorporeal Technology. This has been corrected as follows: "A case study published in CJC Open described a patient who achieved significant gains in VO₂ peak after completing over 1,000 hours of exercise training [35]."

